# Co-designing a prototype teaching intervention on triadic communication for healthcare professionals working with young people with cancer^☆^

**DOI:** 10.1016/j.pecinn.2026.100492

**Published:** 2026-07-14

**Authors:** Deborah J. Critoph, Rachel M. Taylor, Helen Hatcher, Robbie Duschinsky, Lawrence Fayers, Stephanie Smith, Elizabeth Fistein, Luke A.M. Smith, Anna Spathis

**Affiliations:** a Primary Care Unit, Department of Public Health and Primary Care, University of Cambridge, Cambridge, UK; b Department of TYA oncology, Cambridge University Hospitals NHS Foundation Trust, Cambridge, UK; c Cancer Clinical Trials Unit, Cambridge University Hospitals NHS Foundation Trust, Cambridge, UK; d Centre for Nurse, Midwife and Allied Health Profession led Research (CNMAR), University College London and Director of the CNMAR, University College London Hospitals NHS Foundation Trust, London, UK; e Clinical Skills Unit, Cambridge University Hospitals NHS Foundation Trust, Cambridge, UK

**Keywords:** Teaching, Triadic communication, Young people, Cancer, Healthcare professionals, Patients' perspective, Time alone

## Abstract

**Introduction:**

Triadic communication is frequently observed in adolescent and young adult cancer care. Communication is fundamental to quality healthcare and patient-centred care. Little research has focussed on triadic communication specifically in this age group. Importantly professionals find communicating with young people difficult, compounded by limited training opportunities.

**Methods:**

A multi-phase, co-design approach was used to develop a triadic communication educational intervention. The prototype teaching was delivered to twelve multidisciplinary professionals. Feedback on the training experience was gained from semi-structured interviews and analysed using framework analysis.

**Results:**

Virtual blended learning was accessible and engaging. Three key themes were generated. The young person's voice was a core driver to the learners engaging with the teaching. The teaching was considered transformative for learners, particularly gaining insight into the need to give the young person time alone with a healthcare professional, without their supporter. Even within brief virtual teaching, learners wanted an opportunity to practise new skills.

**Conclusion:**

Healthcare professionals valued teaching that enhanced their triadic communication skills. They learnt practical strategies to help them ensure care centred around the young person. The training may be optimised by incorporating experiential elements such as roleplay.

**Innovation:**

We have co-designed innovative triadic teaching for multidisciplinary healthcare professionals. Feedback suggests it was transformative for learners, with some modifications being required. This teaching may be of value in triadic communication beyond adolescent and young adult cancer care and will require further refining and evaluation of the impact on patient outcomes.

## Highlights

• There is a high demand for triadic communication skills teaching.
• The teaching was highly relevant to learners who were invested, and actively engaged.
• Learners felt that they had learnt new skills and wanted an opportunity to practice these.
• A key learning point was the benefit of ‘time alone’ with a trusted healthcare professional.
• Triadic communication skills teaching supports achieving patient centred care.

## 1 Introduction

In the United Kingdom around 2500 adolescents and young adults are diagnosed with cancer (AYAC) annually, and cancer remains the leading cause of non-accidental death in young people globally [1]. They experience long periods of cancer treatment, a worse prognosis than both children and older adults, and a particularly devastating sense of despair and social isolation [2], [3]. Their unique needs have led to an international drive to establish specialist cancer services for young people, delivered by specifically trained healthcare professionals [4], [5], [6].

Research consistently demonstrates how the communication needs of AYACs differ from younger children and older adults. Communication is a research priority for young people, families and professionals, which was highlighted in a research priority setting exercise [7]. Triadic communication refers to the presence of a non-professional third person in clinical encounters, such as a parent, carer or companion, referred to here as a supporter [8], [9]. In AYAC care, communication is typically triadic [10], [11], [12], [13], [14], [15]. Supporters are commonly perceived to be parents, especially mothers, with little known about non-parental supporters [14], [15], [16], [17], [18].

Communication is fundamental to high quality, patient-centred care, attracting much needed research interest [11], [12], [19], [20], [21], [22]. Existing studies have mostly investigated triadic communication involving younger children or older adults [23], [24], [25]. However, little research has considered triadic communication for adolescents and young adults. A recent systematic review evaluating triadic communication in young people with cancer found triadic communication to be prevalent, complex and dynamic [14]. Importantly very little research had considered triadic communication from all three perspectives – young person, healthcare professional and supporter.

Critically, professionals find communicating with AYACs difficult, compounded by limited training opportunities [14], [19]. Existing training tends to be uniprofessional, often ad-hoc, lacking an evidence based and not including triadic communication [12], [19], [21], [26]. Given the limited evidence for both triadic communication and effective multi-professional communication skills training, we undertook a co-design study to develop a prototype teaching intervention to improve communication for young people with cancer. We triangulated experiences to explore the interactional dynamics in this complex communication [27] and utilised the data to co-design the training intervention. Here we share findings from evaluating the delivery of the prototype teaching for multidisciplinary healthcare professionals.

## 2 Methods

**Fig. 1:**
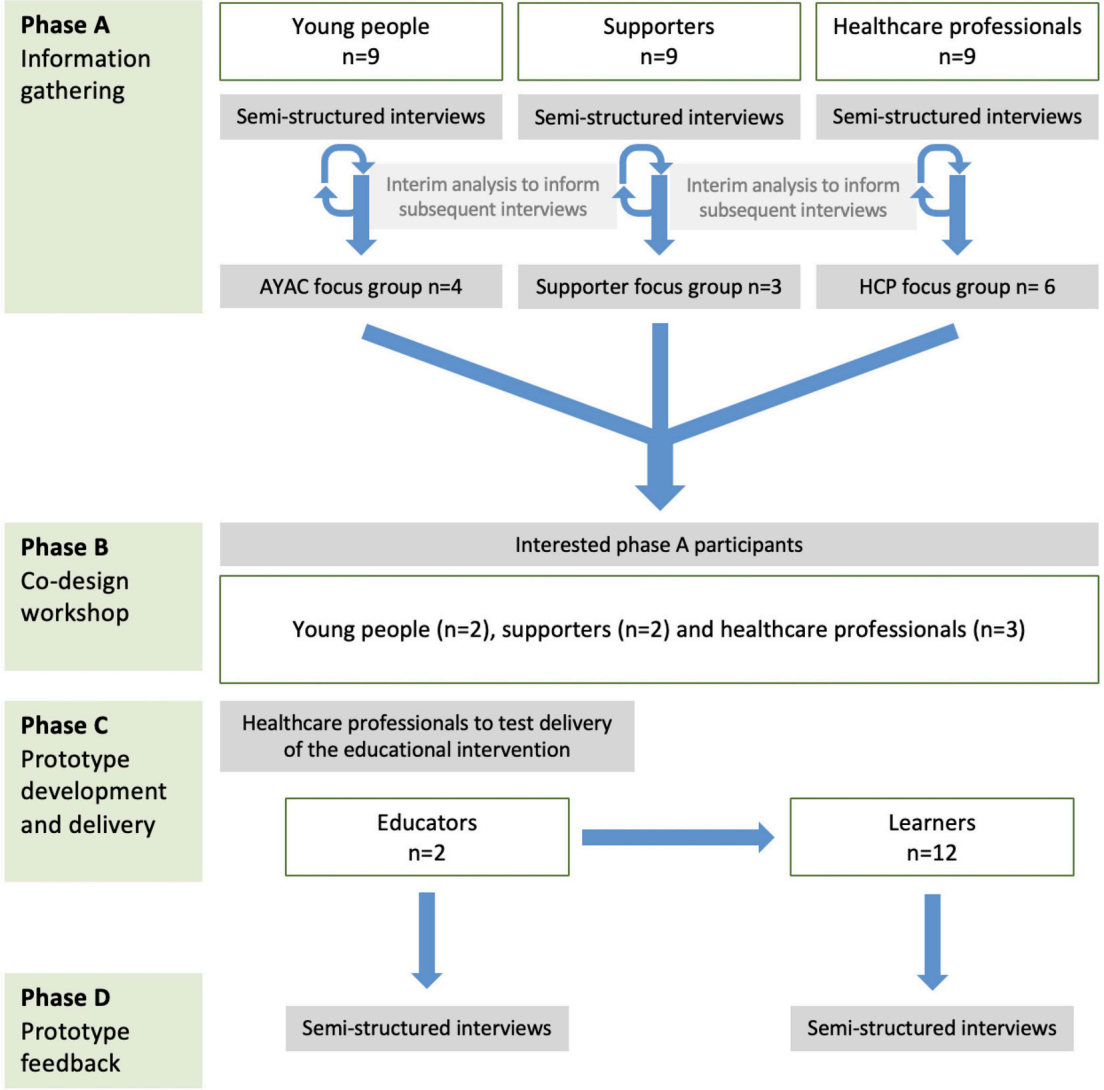
Educational intervention co-design study.

A multi-perspective, multi-phase qualitative co-design study was undertaken (Fig. 1). In Phase A interviews and focus groups were conducted involving all members of the triad. Phase B consisted of the co-design workshop with selected participants from Phase A. Phase C developed and delivered the prototype teaching and Phase D sought feedback from those who received and provided the teaching. This report focuses on the co-design of the prototype teaching and delivery, along with feedback from the learners and teachers. Findings from the initial information gathering phase, examining the dynamics of the triad (Phase A), are published elsewhere. [27]

The findings from Phase A informed the co-design workshop and subsequently the learning outcomes of the prototype teaching, Table 1 [27]. Selected interested participants (*n* = 7) from Phase A and five members of the research team, including the lead patient and public involvement member, attended the virtual co-design workshop lasting 90 min. A presentation detailing suggested learning outcomes, format and teaching methods was delivered by DJC and feedback from all stakeholders was sought. Rich discussion and debate followed. Contentious points included the format (virtual vs in person) and the length.

**Table 1:** Learning outcomes of the prototype teaching.

Aims: To deliver high quality teaching to healthcare professionals, (working with adolescents and young adults with cancer) that gives them the confidence and skills, to be able to navigate differing agendas and identify the young person's perspective in triadic communication encounters, whilst attending to the supporters needs. For healthcare professionals to understand the pivotal role and responsibility that they have in triadic communication to advocate for and hold the young person's wishes central to the conversation.		
Theme	Young person experience	Learning outcomes
Theme 1: Young people frequently become disengaged from the communication triad.	Young people experienced shock, overwhelmed and an inability to listen and engage, exacerbated by poor communication.	To raise awareness of the multiple reasons why young people can be disengaged in triadic communication both internal and external to them, across the cancer experience
	Poor health status also contributed to experiencing inability to communicate entirely or effectively.	Communicate directly to the young person, unless specifically asked to communicate to the supporter
	Feelings of sadness and guilt were experienced from witnessing their supporter's response to the cancer diagnosis.	Elicit the young person's perspective
Theme 2: Non-parent supporters are important to the young person but are sidelined in triadic communication.	Young people have a range of supporters, it should not be assumed that parents are the most important supporter to young people.	To proactively establish who the young person considers to be their main supporter.
	Young people experience communication work to navigate what communication is given to who and when. Parent supporters seek communication from professionals sometimes without permission.	Assess the roles of supporters and establish communication boundaries (to include who is permitted to information from a HCP and who is the documented point of contact), led by the young person. This is particularly important if the main supporter is a non-parent.
	Non-parent supporters were not always the point of contact despite young people requesting this.	Acknowledge the uniqueness of the parent-young adult role. Parents may need guidance to renegotiate their role – regardless of age, they will always be the young person's mother or father
Theme 3: Professionals perceive multi-level threat that collectively hinder triadic communication.	Young people may not experience timely or effective communication because of professionals' level of confidence and perceived skill (as seen in theme 1).	To develop professionals' confidence and skills, to be able to navigate triadic communication encounters, to identify the young person's perspective and wishes, whilst attending to the supporters needs.
	Parent supporters may prohibit communication that the young person receives or may insist on treatment/care the young person did not choose.	Acknowledge the impact of the young person's diagnostic process on their willingness to communicate. Develop professional skills to regain trust of the young person (and supporters).
	Sometimes communication did not adhere to legal frameworks and this limited the young persons autonomy.	To respond to difficult requests from supporters within legal and ethical frameworks.
Theme 4: Tension and conflict are inherent to triadic communication.	Young people did not always have the same agenda or perspective as their supporter or healthcare professional. Young people's threat response was different to a supporter and all three experienced tension.	To develop skills to navigate triadic communication when there are differing agendas (keeping the young person involved to the extent that they want to be)
	Parent supporters did not understand why the young person would need to talk to a healthcare professional alone. Young people wanted one to one time alone.	Know why and how to offer explicit one-to one time alone with a young person without the presence of the supporter

Learning outcomes aligned with the four analytical findings from phase A Critoph DJ, Taylor RM, Hatcher H, Duschinsky R, Fayers L, Smith LAM, et al. Understanding experiences and impact of triadic communication for young people with cancer, supporters and healthcare professionals. A qualitative enquiry. Patient Education and Counseling. 2026;143:109418.

Amendments to the proposed teaching resulting from the co-design workshop included adding video content rather than solely written material into the pre-learning material, and including a patient narrative at the beginning of the teaching. The idea to rename the clinical video vignettes with the young person's needs was also developed in this workshop (“I've got a differing perspective” and “I need time alone with you”).

Hearing all three perspectives of the triad on the suggested intervention was critical to ensure fair representation of all parties in the teaching. The scheme of work (supplemental file 1) was developed based on the outcomes of the co-design workshop. The teaching was grounded in a socio-constructivist framework; this is considered relevant for teaching healthcare professionals because it contextualises learning within clinical practice, and shifts learners from passive learning to active knowledge construction. [28] Resources were created for the teaching intervention, and this included creating clinical video vignettes with professional role players. Guidance was also sought from an expert in Law and Ethics to create content that addressed the learning outcome on communicating within legal and ethical frameworks.

### 2.1 Setting and participants

The educational intervention was delivered virtually using Zoom. All the educators (*n* = 2) and learners (*n* = 12) participated in this research. Learners could be any healthcare professional regularly caring for young people with cancer. It was intentional to offer and test the prototype teaching to interdisciplinary professionals.

### 2.2 Participant recruitment

Two educators were recruited to deliver the teaching. Four experienced clinical communication skills facilitators were approached by DJC, all were willing to participate, and two were selected based on timescales and availability. Twelve learners were recruited nationally through non-governmental organisations specialising in AYAC who circulated adverts via professional networks. There was a notably high level of interest in attending the prototype teaching, resulting in a reserve list of 14 healthcare professionals. Every one of the selected learners attended the teaching.

This study was approved in 2023 by the Yorkshire and The Humber – Leeds West Research and Ethics Committee (23/YH/0152). All participants provided written, informed consent.

### 2.3 Procedure

The learners consented to receive the prototype teaching which included 45 min of pre-learning and a two-hour facilitated teaching session. All learners completed a pre-questionnaire, and a post teaching evaluation (supplemental file-2). Individual semi-structured interviews were conducted with participants to understand the experiences of receiving and delivering the teaching. Interview guides are detailed in Table 2. Author DJC conducted all interviews, which were digitally audio-recorded, transcribed verbatim in English and de-identified. All interviews were conducted virtually using Zoom. Interview mean duration was 28 min (range: 20–48 min).

**Table 2:** semi-structured interview questions for learners and teachers.

*Learners* • Tell me about your experience of being a learner? • What worked well? What didn't work so well? • What would you keep? What would you change? What would you add? • What comments do you have on the length of the teaching and that it was virtual? • Was the learning opportunity helpful for your clinical practice and why? • How will this learning influence your clinical communication - please give as many examples as you can. • How might the impact of this learning opportunity be measurable in your clinical practice? • How could we make this teaching more inclusive, particularly for learners from ethnic minorities or diverse groups, such as those with neurodiversity? • Is there anything that you may have been expecting that was missing? • What comments do you have on the resources that accompanied the educational opportunity? • Was 45 min acceptable? • We aspire to roll this out nationally – what comments do you have on this? • Please feel free to add any additional comments. *Teachers* • Tell me about your experience of delivering the educational intervention to a group of learners? • What worked well? What didn't work so well? • What would you keep? What would you change? What would you add? • Do you feel the teaching was helpful for the learners? If yes, why? If no, why not? • Do you feel that you have learnt something that will influence your own clinical/teaching practice? • How could we make this teaching more inclusive, particularly for learners from ethnic minorities or diverse groups, such as those with neurodiversity? • Did you have enough information to deliver the educational intervention? • Did you have the knowledge and skills to deliver this intervention? • What else would have been helpful to prepare you to deliver the education? • What else would you need to deliver this educational intervention again in the future?

### 2.4 Data analysis

Data were analysed using framework analysis in five stages: familiarisation, indexing, charting, mapping and interpretation [29]. The transparency of framework analysis enabled multiple team members to review the findings. The coding framework was independently checked by another researcher (RMT) who part coded the data (four transcripts) using a consensus-based approach to resolve disagreements and develop a shared understanding of the coding framework. When consensus had been established and the framework was refined, DJC coded the remaining data set.

Whilst DJC and RMT coded, HH, AS, RD, and LS had oversight of the analysis and were part of the analytical and interpretive discussions. Integration of perspectives was helped by discussion. NVivo 14 software was used to support data management. Themes and sub-themes were defined and refined iteratively through multiple consensus discussions, member checking and debriefing within the entire research team. This included revising the analysis to confirm there was detailed but not overinterpretation. The researchers engaged in reflexivity, acknowledging their professional and scientific roles, attitudes and biases, and looked for deviant cases.

### 2.5 Patient and public involvement

Two young people, with a past cancer diagnosis and extensive relevant lived experience, were key partners in this research from it's inception. Their roles included reviewing all participant facing materials. One member attended research activities, provided feedback on the early findings and was involved in data analysis. Their contributions significantly shaped the direction of this work.

## 3 Results

### 3.1 Participant characteristics

The two educators, of different genders and disciplines, regularly teach clinical communication skills in a variety of settings to undergraduate and post graduate multiprofessionals.

A multiprofessional group of 12 learners participated in the teaching including registered nurses (*n* = 6), allied health professionals (*n* = 3) (dietician, physiotherapist and occupational therapist) and doctors (*n* = 3). All learners provide care regularly to AYAC. Eleven were female with one male learner.

### 3.2 Prototype teaching format

This was a two-hour facilitated, virtual teaching with six learners at a time. The teaching was delivered on two occasions. Pre-learning materials (45 min) were sent 2 weeks in advance. The teaching combined multiple teaching methods to promote learning through problem solving and engagement with each other (Table 3). The full scheme of work is in supplementary material file 1.

**Table 3:** Prototype teaching format.

Empowering young people in triadic communication – virtual teaching for healthcare professionals caring for young people with cancer.	
Pre-learning	Short video: overview of triadic communication
Total 45 min	Short video: overview of ethical principes.
	Read ethical case scenario: request to withhold information.
Young person perspective	Patient narrative video. Learners to comment in the chat function on what surprised them and what they were curious about. Followed by group discussion.
Small group work in break out rooms	Unfacilitated discussion occurred in two breakout rooms on ‘how supporters may behave in a triadic communication encounter’. This was followed by 5-min whole group didactic teaching to consolidate learning.
*Comfort Break*	
Clinical video vignette “I've got a different perspective”	The objective here was to develop skills to navigate triadic communication when there are differing agendas Learners observed 2 videos and invited to comment: What did they notice? What would they do differently?
	Consolidated with teacher slides - core triadic skills.
Ethical Case Study	Two groups of three to discuss: how could you respond to the mothers request not to tell her daughter she was dying.
	Consolidated with teacher slides of key points.
Clinical video vignette “I need to be alone with you”	The objective here was to consider how to offer explicit one to one time alone with a trusted healthcare professional
	Learners observed 2 videos and invited to comment: What did they notice? What would they do differently?
	Consolidated with teacher slides navigating time alone.

### 3.3 Themes

Three broad themes were identified. Exemplifying quotes for each theme are provided as follows; (AHP – Allied healthcare professional, DR – Doctor, RN- Registered nurse T- Teacher).

#### 3.3.1 The young person's voice is a core driver for engagement

The format of the prototype teaching was accessible and acceptable to the learners, with the patient's voice considered particularly impactful. Learners were highly engaged throughout the teaching, with the patient narrative, case study and clinical vignettes being crucial to this. The patient narrative allowed learners to ‘hear’ the young person's experiences of triadic communication across the cancer experience; actively contributing reflections, as they listened, heightened their engagement. The legal and ethical case study encouraged learners to problem-solve how they would respond to a mother's request to withhold information. Learners valued gaining early access to the case study within the pre-learning as it gave them time to consider this complex challenge before discussing as a group.

*“I thought the case study worked really well. The girl talking about her experiences that was really helpful to set the scene, and make you reflect on similar discussions that I might have had”*

AHP1

“*I like that, having the chat open and me typing while I was watching the video, I've never done that before. I thought that was really good, because it did make me watch*”

RN2

The clinical video vignettes focussed on how to navigate different perspectives in the triad and how to offer explicit and confidential one-to-one time alone. These were considered thought provoking and engaging. When asked what was missing most learners had no suggestions, though a few expressed a desire for more patient narratives. One learner said, “the more we see and hear, the better we will be” and another suggested a wider variety of ages may have been beneficial.

“*Actually, seeing those videos and the young people's perspective, that kind of hits home a little bit harder than just talking about it*”

AHP3

“*It was nice that we saw the videos of sort of what not to do and then we broke it down in groups and were then able to look at how it could have been done with recommendations*”

AHP1

Several other factors drove engagement with the teaching. Although most learners had an inherent preference for in-person teaching, many acknowledged that virtual formats were feasible, familiar and effective. The small group size, ‘chat function’ and use of breakout rooms enhanced interactivity and contributed to their engagement. Participants particularly valued the opportunity to learn with a national multi-professional group. Attendees were drawn from across the UK, facilitated by the remote delivery. A minority of learners experienced technical difficulties and switched from laptops to telephones.

“*What worked well was the group size, I think small group size is the right thing*”

RN4

“*Breakout rooms with the opportunity to talk and listen was really good*”

RN1

Many expressed surprise at the effectiveness of the virtual teaching. The clear instructions to learners to keep their cameras turned on in the virtual environment was valued making it more ‘personal’. Learners enjoyed the variety of teaching methods and the expectation to contribute often. Many learners described a safe learning environment and felt empowered to contribute due to the facilitator finding ways of everyone contributing; one learner commented it was “clever getting everyone to speak”.

“*It was a nice shake-up with open discussion, videos, so it was not all slide, slide, slide, it was a nice mixture of discussion and visual aids as well as the alternate videos as well in the breakout rooms. So, I found it was one of the better teachings that I've had and it was clear, so I enjoyed it*”

RN3

The pre-learning was acceptable and prioritised by learners, with 11/12 completing it. Participants found it a good introduction to triadic communication, and valued that it did not reduce the interactive teaching time. It was noted engagement in the case study without the legal and ethical video presentation would have been difficult. One educator questioned whether the pre-learning felt too separate from the live teaching, although learners generally found the narrated PowerPoints engaging and integral to the session.

*“I thought the pre-training was really good and really helpful and a necessity, really, before you did the 2-hour face-to-face training”*

AHP1

#### 3.3.2 Even short on-line teaching may benefit from including an experiential component

The need for triadic communication teaching was far greater than anticipated, with learners wanting both longer teaching and, particularly, an opportunity to practice what they had learnt. Previous communication training had focused on dyadic communication with either children or adults. Several learners noted limited availability of training focused on adolescents and young adults, and they were excited to receive age-specific training. They valued receiving triadic communication support and gaining insight into all three perspectives within the triad.

*“I've done advanced communication skills based on two-way communication and I think, over time, with experience, you learn what works well and what doesn't work well in triadic communication without necessarily having so much of a framework and, you know, a theoretical framework works well”*

DR2

*“The real positive was seeing things from each perspective of that triadic communication and kind of making allowances for each of the people involved in that. Recognising the difficulties of it”*

RN1

Many learners would have valued longer teaching allowing more time for in-depth discussion and an opportunity to practice. Most wanted a whole day; learners reflected you could have “a whole day talking about it” and “there was a full day there of material”. These learners would prioritise a day. For the minority, 2 h was the maximum they could schedule. One learner commented that the timing felt ‘perfect’ though by the end wished the teaching had been longer.

*“It was actually much shorter than I thought it would be, it was really short, but actually kind of probably the right length in that it was enough information to learn lots but not be overwhelmed, and then come away and be like, it was brilliant”*

RN 4

*“I'm a big advocate for a full day, so you get the best out of the teaching session”*

RN6

A few learners felt time for discussion in group work was limited, especially in the ethical case study. However, learners did not feel rushed. Conversely both of the educators felt rushed and under pressure which one commented was stressful. Teachers felt they were cutting short rich discussion that could have been extended. Participants summarised that a minimum of an additional 30 min would be useful.

*“I think it would have been better had it been longer because there was so much that I really wanted to kind of hear more about and just develop a bit further”*

RN1

“*We could have done more of it, it did feel rushed, I felt there was times where discussion could have been extended, and could have brought such rich sharing of experiences, and I felt that we were shutting that down quite quickly*”

T2

Learners consistently commented on the lack of opportunity to practice the skills they perceived they had learnt, although a few felt confident to try new skills with patients without prior practice. Some learners said they loved roleplay and would have valued it. One learner was relieved roleplay was not included because of a negative experience. Another learner who disliked roleplay reflected it would have been beneficial. The educators unanimously felt the teaching would have benefitted from opportunity to include roleplay; learners would experience the emotions; they would get to “mess up” in a safe environment. One educator reflected “the level of confidence people have in discussions is different to their behaviour of putting it into practice”. Both educators shared a bias that experiential learning was the most effective way of teaching communication skills.

*“We could do like roleplays and actually feel like we're applying what we've learnt from the session into practice whilst we're in that safe learning environment before we go back into our day-to-day jobs and try and apply those skills in the real world”*

DR3

“*Particularly how do you phrase time alone, being able to practice that, you know, listening to it, discussing it is one thing, actually facing somebody for the first time doing that, better for it to be with an actor, than it is with a real parent”*

T1

#### 3.3.3 ‘Time alone’ is a threshold concept in triadic communication teaching

All learners reflected on how relevant the teaching was to their own clinical practice, one learner noting, for example, “a lot of the themes resonated with me”. Many learners appreciated gaining insights into the nuanced impact of the presence of the supporter within a communication encounter. The learners were candid about the challenges of triadic communication, with one learner reflecting that it is “just profoundly difficult”. For many, it raised their awareness and phrases “eye opening”, “it made me think” and “it was like a lightbulb” were commonly used as the learners actively engaged in the teaching.

Many learners described their realisation that the young person could be central within the communication, with a need for one-to-one ‘time alone’ with the young person and healthcare professional. Learners began to recognise the extent to which they were allowing the supporter to steer the conversation. They gained insight into the impact of the supporter on the communication, and how pivotal a professional could be in navigating the encounter to ensure the young person's perspective was elicited. Several learners said they learnt practical strategies to recentre the communication encounter.

*“It increases my anxiety having a parent there because it's as if a peer is watching you and analysing your performance while you're trying to get to know the young person as well, it just feels like it's another level of communication skills*”

AHP1

*“I guess it heightens awareness of what might be going on in that consultation that maybe you weren't quite so aware of before”*

RN5

*“Sometimes I know that I might let the third person talk over them or talk too much, that actually made me think, you can tell them, ‘Hang on one minute’ and redirect the questions”.*

AHP2

Most participants felt these insights were transformative, changing their way of thinking about triadic communication encounters. Giving young people ‘time alone’ emerged as an educational threshold concept. These learners appeared genuinely surprised when asked about time alone, suggesting significant gaps in their understanding. It was acknowledged by learners that there had never been any teaching on how “how to do it practically or about the benefit of it”. After the teaching, learners felt more confident offering confidential one-to-one time alone.

Only one learner (a paediatric clinician) shared concern regarding time alone. They worked in a hospital where a professional was convicted of paedophilia “everything that's been setup from a Trust [hospital] perspective has been to prevent one-to-one conversations from happening”. This learner felt motivated to consider ways in which this could be overcome having realised the benefits for this age group.

*“I've got a summary on my diary, ask them to introduce who's in the room, set the agenda and to discuss the time alone as a routine part of our interventions, just to, just to keep me, with every interaction”*

AHP1

“*Some of the specific themes about interactions and offering the one-to-one time, were really helpful making the confidentiality side and saying, ‘Look, it's confidential, but it isn't, in the sense that there are some things that might come up,’ it was done in a really kind and supportive way and that bit's always the really difficult bit, and that's something I've learnt from that which I can take away into my own practice*”

DR2

“*I think I'll definitely been more confident about asking the parent, the supporter to step out or to be on their own, again, because it's not something that feels easy*”

RN2

The prototype teaching was considered to have high transferability to other clinical areas, “it could be bigger than cancer”. One learner reflected “As someone with a lot of experience, I thought I'm not going to learn anything new, I think it'll be interesting, but I really did”. Learners concluded that anyone working in AYAC should receive this teaching, it could be mandated training and rolled out nationally. It was also deemed appropriate for professionals outside of oncology, such as primary or emergency care.

*“I think it would be really good for everybody to do it. Just even consider how to manage it. I think it needs to be longer and everywhere”*

RN1

*“I think there's definitely a gap that needs to be filled and can be filled, and would benefit lots of people, I think that would be hugely beneficial”*

RN5

## 4 Discussion and conclusion

### 4.1 Discussion

This research identifies a gap in teaching provision and proposes ‘how’ to teach triadic communication skills. The results align with wider literature demonstrating professionals find triadic communication challenging and this is not routinely taught [14], [20], [27], [30]. This was a diverse group of professionals with varying levels of experience in AYAC care where attendance was optional. The high demand (and waiting list) to attend this teaching highlights the great need from professionals for triadic communication teaching.

The teaching was grounded in a socio-constructivist framework, which is a process where learners construct knowledge socially through language, tools and participation in activities requiring critical thinking [31]. This ‘new’ knowledge is integrated into prior schemata to develop concepts and skills beyond what they are capable of doing alone with the community of healthcare practitioners [28]. This view of learning posits that learners co-construct understanding through interaction and collaboration and recognises learners' prior knowledge. These learners were all post-graduate professionals working in AYAC with experiences of triadic communication.

The teaching consciously used real clinical contexts of triadic communication [27]. Socio-constructivism and socio-cultural learning worked together by using real contexts as the environment for learners to actively build their own understanding [32]. The teaching methods were aligned with socio-constructivism and socio-cultural learning theory (to scaffold understanding) through co-construction in interaction with the AYAC community of practitioners, using clinical cases generated from the qualitative information gathering phase of this work in Phase A [27]. The results show the teaching was highly relevant to clinical practice and context which enhanced learning in this intervention. Participants valued the multi-professional perspectives, fostering greater breadth and depth of learning.

Pedagogical interventions were deliberately chosen to promote active involvement and aligned with social constructivism [28]. This was successful as learners reflected that they felt themselves become actively engaged in the learning allowing for deeper learning. This was enabled by highly skilled, clear facilitation, noted by the learners. The facilitators created safety in the group, this is critical because the educational environment has a major impact on promotion of effective learning. This type of learning can create judgement, threatening professional identity. There were two instances in unfacilitated breakout rooms where learners felt dismissed. The literature supports the value of small group sizes, which can optimise individuals making sense of the teaching and co-constructing meaning [28], [33].

The results showed that the patient narrative, case study and clinical video vignettes were particularly powerful learning components. These elements engaged learners emotionally and intellectually, making the material more memorable [34]. These interventions were used actively, with participants problem solving collaboratively to create solutions, rather than memorise facts. The problem-based learning approach was effective for many learners. The opportunity to consider triadic communication in compelling formats enabled the learners to “reflect on action” [35]. Many learners said “It made me think” allowing deeper consideration of these complex dynamics that they had not previously considered.

The length of the teaching is a dilemma as an educator. The 2 h was accessible for all and learning was evident. However the teaching did not provide an opportunity for learners to practise the strategies discussed. This was a deliberate decision to keep the intervention brief but the results clearly show that most learners wanted the chance to practise and receive feedback. This was also the view of the teachers and is the gold standard for developing communication skills [36], [37]. Brown argues that unless learners are given the opportunity to practice in the classroom the learners will struggle to transfer the learning to the clinical environment [38].

Many learners wanted to be pushed beyond the zones of proximal development and could not do this without practice and feedback from more knowledgeable others [28]. Many learners specifically wanted to practice negotiating time alone. Time alone with a trusted healthcare professional can facilitate exploration of the patient's perspective and is considered a cornerstone of quality adolescent healthcare [14], [27], [39], [40], [41], [42], [43], [44]. Previous reports describe professionals lacking the confidence and skill needed to achieve time alone with their young patients [40], [45]. The findings in this study are consistent with this, and move the literature forward by finding ways of supporting healthcare professionals to gain these skills.

The translation of learning into clinical practice would likely have been greater if the intervention had included this practical component. There is a tension between shorter, accessible training interventions which raise awareness of triadic communication and longer sessions allowing deeper learning with opportunities to practise skills in order to increase confidence and likelihood of practice change.

### 4.2 Innovation

There is a clear clinical and educational need to teach triadic communication to healthcare professionals involved in young adult cancer care and this has not previously been included in teaching. This teaching innovation was co-designed with all stakeholders including strong PPI and has been developed and tested. The findings suggest that the prototype intervention offered a useful foundation for developing these complex communication skills; however, modifications are required. The trial-ready intervention will deliver this core teaching in a slightly longer three-hour virtual format. Learners will then be offered the opportunity to attend an optional full-day in-person session incorporating experiential learning with role-play to practise new communication skills. This would be delivered at three locations across the UK to minimise geographical barriers to participation.

The difficulties professionals face in communication is evidenced, and this teaching gave the learners practical strategies with which to navigate these. The content was of clinical relevance because it was evidence based and co-designed with all stakeholders including professionals with whom the teaching was aimed at. Of great relevance, learners learnt ways to hold the young person central and illicit the patients perspective, without which patient-centered care cannot be realised. Future research in a clinical trial is required to determine if this teaching has an impact on patient care.

### 4.3 Strengths and limitations

This study has triangulated perspectives to generate an effective and well received teaching intervention. The co-design process has generated a prototype teaching considered highly relevant by participants. Teaching was delivered to a diverse range of national healthcare professionals in a variety of multi-professional roles. Participants all spoke English and did not have any additional learning needs, therefore we do not understand the nuances of teaching triadic communication in other language and sociocultural contexts or to those with additional needs. The sample size was relatively small due to the nature of the prototype teaching intervention and these professionals all had experience in AYAC care. It is not known whether this teaching would be relevant for professionals involved in triadic communication out of an AYAC context.

### 4.4 Conclusion

There was an unanticipated high demand from healthcare professionals to attend the triadic communication teaching. The teaching was enjoyed and found to be highly engaging and accessible to learners in a virtual environment. The learning was of relevance and resulted in professionals having clear practical strategies they could implement to improve triadic communication that holds the young person central to the communication encounter. Transfer by professionals of the learning to clinical practice, may have been enhanced if the teaching had included experiential elements such as roleplay given the complexity of this communication. The impact of the revised intervention on patient care will subsequently be tested in a clinical trial with professionals inside and outside of oncology.

## CRediT authorship contribution statement

**Deborah J. Critoph:** Writing – review & editing, Writing – original draft, Visualization, Validation, Software, Resources, Project administration, Methodology, Investigation, Funding acquisition, Formal analysis, Data curation, Conceptualization. **Rachel M. Taylor:** Writing – review & editing, Visualization, Supervision, Methodology, Investigation, Formal analysis, Conceptualization. **Helen Hatcher:** Writing – review & editing, Supervision, Methodology, Investigation, Formal analysis, Conceptualization. **Robbie Duschinsky:** Writing – review & editing, Supervision, Methodology, Investigation, Formal analysis, Conceptualization. **Lawrence Fayers:** Writing – review & editing, Formal analysis, Conceptualization. **Stephanie Smith:** Writing – review & editing, Visualization, Methodology, Conceptualization. **Elizabeth Fistein:** Writing – review & editing, Supervision, Resources, Formal analysis. **Luke A.M. Smith:** Writing – review & editing, Resources, Methodology, Formal analysis, Data curation, Conceptualization. **Anna Spathis:** Writing – review & editing, Supervision, Methodology, Investigation, Formal analysis, Conceptualization.

## Declaration of competing interest

The authors declare the following financial interests/personal relationships which may be considered as potential competing interests:

Deborah Joy Critoph reports financial support was provided by Wellcome Trust. Deborah Critoph reports a relationship with Wellcome Trust that includes: funding grants. If there are other authors, they declare that they have no known competing financial interests or personal relationships that could have appeared to influence the work reported in this paper.

## Data Availability

Due to the potentially sensitive nature of interview discussions, our ethical approval does not allow public sharing of the qualitative data set that forms the basis of this report. However, on request by e-mail to the corresponding author, the anonymised data can be made available.

## Appendix A Supplementary data

Supplementary Material S1 Prototype teaching scheme of work.

Supplementary Material S2 Pre learning questionnaire and immediate teaching evaluation.

Supplementary Material S3 COREQ checklist

Supplementary Material S4 CRe-DEPTH checklist
