# Supplementary material for: Co-designing a prototype teaching intervention on triadic communication for healthcare professionals working with young people with cancer
Source: PEC Innov. 2026 Jul 14;9:100492. doi: 10.1016/j.pecinn.2026.100492 (PMC13449821; doi:10.1016/j.pecinn.2026.100492)
Supplement: Supplementary file 1 [file mmc1.pdf]

UNIVERSITY OF CAMBRIDGE

Scheme of work – Triadic communication in teenage and young adult cancer care

Empowering young people in triadic communication – virtual teaching for health professionals caring for young people with cancer.

The prototype intervention will be delivered in two small groups with a maximum of six learners in each group – one facilitator per group.

Non-participating observers will be present. They will be introduced to the learners and then have cameras off throughout and remain silent.

Description
This will be delivered on-line for two hours of teaching. It outlines background, session materials and scheme of work. The scheme of work includes: the timetable for the learning with details of activities/exercises with associated objectives.

Background
This educational intervention (teaching) is for healthcare professionals (HCP) working with teenagers and young adults (TYA) with cancer (TYAC).

Pre-session work for participants:

The total time for pre-session work is 45 minutes.

1. Watch a narrated presentation to set the scene by Deborah Critoph. (The final slide will include key references listed on page 2 should the learners want to access them) 19 minutes.
2. Watch a recorded lecture by the law and ethics lead Dr Elizabeth Fistein summarising overarching ethical principles for 13–25-year-olds. 21 minutes.
3. Case scenario – given ahead to give the learners ample opportunity to consider communication following a difficult request to withhold information from a mother.

Key learning objectives
**Aim:** To deliver high quality teaching to healthcare professionals, (working with teenagers and young adults with cancer) that gives them the confidence and skills, to be able to navigate differing agendas and identify the young person's perspective in triadic communication encounters, whilst attending to the supporters needs. For healthcare professionals to understand the pivotal role and responsibility that they have in triadic communication to advocate for and hold the young person's wishes central to the conversation.<br><br>**Learning Objectives:**<br><br>1. To understand the reasons why young people can become disengaged from the communication triad.<br>2. To proactively establish who the young person considers to be their main supporter and establish communication boundaries led by the young person.<br>3. To develop skills to navigate triadic communication when there are differing agendas, keeping the young person involved to the extent that they want to be.<br>4. To be able to respond to difficult requests from supporters ethically.<br>5. To consider ways of offering explicit one to one time with a trusted healthcare professional (time alone)

Key references:
**Further reading:**<br><br>TYAC Good practice guideline on communicating with teenagers and young adults with cancer – accessible here: https://www.tyac.org.uk/tyac-good-practice-guides/communicating-with-teenagers-and-young-adults-with-cancer.<br><br>Critoph, D, Cable, M, Farmer, J, Hatcher, H, Taylor, R and Smith, L (2024) Is There Scope to Do Better? Clinical Communication with Adolescents and Young Adults with Cancer – A Scoping Review. Psycho-Oncology. **doi: 10.1002/pon.6317**<br><br>Critoph, D, Taylor, R, Spathis, A, Duschinksy, R, Hatcher, H, Clyne, E, Kuhn, I and Smith, L (2024) Triadic communication with teenagers and young adults with cancer: a systematic literature review – 'Make me feel like I'm not the third person'. BMJ Open. **doi:10.1136/ bmjopen-2023-080024**<br><br>Silverman JD, Kurtz SM, Draper J (2013) *Skills for Communicating with Patients* 3rd Edition, Radcliffe Publishing, London.

Session Materials:

1. Pre-course materials, sent to learners four weeks ahead of the day. These are as listed above; total time is forty-five minutes. The resources will be on u-tube and learners will access using the link.
2. A welcome letter with detailed instructions, working agreement, aims and objectives, and outline of the day will also be sent four weeks ahead. Learners will be asked at this point if they have any learning needs or accommodations that the facilitators need to be aware of.
3. Evaluation form to be completed at the end of the session – this will be an on-line evaluation form that can be accessed via a QR code by the learners at the end. Facilitators to please encourage that learners stay in the zoom environment until they have completed this form.

Mode of delivery:

1. The teaching will be delivered on-line, using the platform Zoom, from a premium university account that will not cut out after 45 minutes.
2. Learners will have received, in the welcome letter, detailed instructions about learner expectations in the on-line learning environment.
3. The teaching includes multiple modes of delivery that include patient narrative, didactic presentation, group work, paired work, and video vignettes.

Deborah Critoph (Lead Researcher) will:
Take overall responsibility for recruitment and communication with learners and send the welcome letter. From this point DC will liaise with learners if they e-mail with any learning needs or questions. Certificate of attendance will be provided by DC.

UNIVERSITY OF CAMBRIDGE

Timing	Objectives	Exercises to meet set objectives
**08.45**	Ensure that the online environment is ready and working as required.	Check all session materials (PowerPoint, videos including sound, details of learners). Put name and facilitator on your video window.
**08.50**	Registration	Open the zoom learning environment and welcome participants into the learning space. Build rapport, gently encourage everyone to write their name and role on the video.
**09.00-09.10**	Introductions, housekeeping and outline plan of the 2-hour session<br><br>Build rapport with the group<br><br>Learner safety	Introductions (Slide 2):<br>Facilitator to introduce self – name, role and specialist interest in this subject<br>Participants introduce self – name, role, where they work, number of years' experience working with TYAC.<br><br>Housekeeping has been covered in the welcome letter.<br>You may want to gently remind learners of the following:<br>Zoom expectations – camera and sound on, confidential space. Engage and contribute as much as possible. One break for ten mins. Encourage questions in the chat. No question is a silly question.
**09.10-09.30**	To understand the reasons why young people can become disengaged from the communication triad.<br><br>**Title: Why young people can become disengaged.**<br><br>**LO 1**	Patient narrative<br><br>• A young person (GK) will give her experiences of communication and the consequences of, in this short twelve-minute recording (slide 4)<br>• Ask learners to write in the chat up to 3 things that you have noticed that you had not previously considered actively listening to the young person's narrative – do this while you are watching the video (slide 3)<br>• Facilitator – please draw attention to the dynamic nature of the communication – the communication preferences, emotion, ability did not stay the same over the treatment trajectory – G does this well in her 12 minute video (slide 4)<br>• Conclude using slides 5 and 6 with the number of ways a young person is disengaged from the triadic encounter.<br>• "Don't take silence as a proof of understanding" Smith et al (2020)

		Key learning points for clinicians (slide 5,6 and 7)<br><br>• Internal reasons why young people are disengaged<br>• External reasons why young people are disengaged<br>• Triadic communication is not static, and healthcare professionals must be flexible and adaptable.<br>• Acknowledge the need to frequently reassess YP communication preferences as they change continually across the cancer experience.<br>• Develop skills to reassess communication preferences and core communication skills to empower YP to speak and be heard.<br><br>Slide 7 is a summary – key considerations slide.
**09.30-09.50**	To proactively establish who the young person considers to be their main supporter and establish communication boundaries led by the young person.<br><br>**Title: Talk to me.**<br><br>**LO 2**	**Triadic communication**<br>Overview given in the pre-learning presentation.<br><br>Supporters – who are the supporters also covered in the pre-learning presentation.<br><br>**Groupwork:**<br>In 2 groups discuss – what do supporters do (parental and non-parental) in a communication encounter. You may want to divide into helpful (facilitative) and less helpful (blocking). Slide 8.<br><br>Ask each group to share two things. Consolidate with slide 9..<br><br>Key learning points for clinicians (slide 10)<br>• Establish who the YP considers to be their main supporter.<br><br>• Assess the roles of supporters and establish communication boundaries (to include who is permitted to information from a HCP and who is the documented point of contact), led by the young person. This is particularly important if the main supporter is a non-parent and must include consent for the third person to be present.

		- Acknowledge the uniqueness of the parent-young adult role. Parents may need guidance to renegotiate their role – regardless of age, they will always be the young person's mother or father. - Involve non-parental supporters.
**09.50-10.00**	Coffee Break	Facilitator to remain in the space for questions.
**10.00-10.15**	To develop skills to navigate triadic communication when there are differing agendas (keeping the young person involved to the extent that they want to be)<br><br>**Video vignettes – titled "I've got a different perspective"**<br><br>**LO 3**	Video Vignette – **"I've got a different perspective"**<br>*Please highlight that these vignettes are not good or bad, they are raw materials that give us something to reflect on and allow us to all recall key learning points. Slide12.*<br><br>**Show version 1** (VB) – ask 2 people to observe (on the outside) the healthcare professionals, 2 to view Olly and 2 to view Helen. Share in the chat what they are noticing. In relation to the objective of keeping the young person being involved.<br>When video is ended – ask for suggestions for change.<br>**Video length – 2 mins**<br><br>**Show version 2** (VG) – ask 2 people to view the healthcare professionals, 2 to view Olly and 2 to view Helen. Share in the chat what they are noticing<br>**Video length – 3 mins 20 secs**<br><br>Facilitator – summarise core triadic communication skills, this is also supported on slide 16.
**10.15-10.30**	To be able to respond to difficult requests from supporters ethically.<br><br>**Titled – HCP need training.**<br><br>**LO 4**	There are two components to this.<br>*Learners will have viewed a recorded lecture by the law and ethics lead with overarching ethical principles for 13–25-year-olds. The activity below is not dependent on having watched this although it will help significantly. If learners have not watched it, please encourage them to view it after the teaching to consolidate learning.*<br><br>Firstly – Group work in two small groups.<br><br>Case example – learners have been sent this ahead of the teaching:

		Group discussion for 10 minutes. Facilitator will summarise key points and offer suggestions (as one example – not the only way). Slides 20 and 21.
**10.30-10.50**	To know how to offer explicit time alone with young people one to one with a trusted healthcare professional.<br><br>**Video vignette titled – "I need to be alone with you"**<br><br>**LO 5**	Video Vignette – **"I need to be alone with you"**<br><br>Present 2 slides – why this is important to young people (slide 21) and what may make it tricky (slide 22)<br><br>This is Alex and mum Alison.<br>*Please highlight that these vignettes are not good or bad, they are raw materials that give us something to reflect on and allow us to all recall key learning points. Slide 23.*<br><br>**Version 1** (VB) – ask learners to write in the chat what they think the impact was and what suggestions for change that they have.<br>**Video length 0.50 seconds**<br><br>**Version 2** (G) – observe how time alone is signposted too.<br>**Video length 02.37 seconds**<br><br>Facilitator – summarise core principles for time alone. Key learning is that young people want HCP to offer this. Slides 26 and 27.
**10.50-11.00**	**Closing**	Thank all learners for engagement and time. Evaluation form using QR code and signpost to certificate to be sent and semi-structured interview with lead researcher Debbie Critoph will follow as per schedule.<br><br>Please stay online if there are questions. Keep a note of these questions and the time that the learners wanted with you.

**Further reading:**
➢ Observe communication with young people in clinical practice<br>➢ Signpost to extended learning references<br>➢ Practice some of the skills that you have learnt within this session as soon as possible<br>➢ MeFirst resources<br>➢ Calgary Cambridge Guide to communicating with patients<br>➢ HEEADSSS assessment

<u>Extended Learning/References</u> – Read the article below:
Bourdeanu, L and Cannistraci, P (2018) Challenges and Role Changes in Caring for Adult Children with Cancer. Advanced Practice Oncology, Sep-Oct; 9 (6): 634-638.

<u>Extended Learning</u> – Link to Clinical Ethics Committee resources and UKCEN website and IME website. Moral distress pocket card (very useful) and British Medical Association Ethics toolkit, children and young people

<u>Extended Learning</u> – Read the article below:
A Ward, Critoph, D, Westacott, R, Williams, R and Dogra, N (2022) A collaboration on teaching and assessing triadic consultation skills.PECInnovation.doi.org/10/1016/j.pecinn.2022.100091
