# Supplementary material for: Co-designing a prototype teaching intervention on triadic communication for healthcare professionals working with young people with cancer
Source: PEC Innov. 2026 Jul 14;9:100492. doi: 10.1016/j.pecinn.2026.100492 (PMC13449821; doi:10.1016/j.pecinn.2026.100492)
Supplement: Supplementary file 2 [file mmc2.docx]

Pre-learning Questionnaire Results

Twelve out of twelve responses were received. 100% response rate.

1. What is your professional role?

2. How confident are you communicating with a teenager or young adult with cancer when a third person (supporter is with them?)

3. What communication skills training have you previously received, and did it include triadic communication?

Advanced communication skills

Nil specific, mainly based on experience

Psychological training level 2

Communication skills as undergraduate; advanced communication skills as postgraduate (Dr); no formal triadic communication skills training – developed organically over time from substantial experience.

Sage and Thyme, Macmillan advanced communication skills

Advanced communication skills. Some triadic skills I seem to remember, but as I am a children’s nurse and previously only nursed children it was something I was very used to.

Advanced communication course

Advanced communication skills (3-day Maguire) and “Enhanced communication skills” (2 days)

I studied Medicine at the University of Cambridge and have therefore undertaken the clinical communication skills module as part of the course

Me first course: Children and young people centred communication

Advanced communications course. Did not include triadic communication.

Advanced communication skills (a long time ago). No, it didn’t include triadic communication.

4. What do you find difficult about triadic communication?

Parents/supporters taking over control of the discussion

Often parents like to do the communication and TYAC may be passive. I try to engage both.

Trying to get a young person to talk when parents are speaking on their behalf. Helping both young person and third person to be able to speak openly about how they are feeling finding things, without trying to protect the other person.

Overbearing third person (s)

People, especially parents, talking on behalf of the patient and not listening enough

Now that I am working with adults it is shifting my focus away from the parent onto the young person more

Speaking to the young patient with the parent trying to intervene and speak on behalf in a very difficult complex discussion

Ensuring the young person is felt heard whilst being respectful to the parents who sometimes want to talk over the young person

I sometimes find it difficult to ensure that both the patient and third person feel listened to, particularly if one is a lot more vocal than the other.

Overpowering parents, disengaged patient

Trying to politely let the parent/significant other to let the YP speak

Sometimes difficult to engage the young adult when a parent/carer/supporter is there if speaking on their behalf. Sometimes difficult to assess how the young person is feeling about the situation, making the choices they want to choose etc.

5. What are you hoping to learn in this teaching?

Improve understanding and communication

Different techniques or strategies for communication

Tools to overcome the things I find difficult

Confirm effective current practice developed organically; identify new practices that will facilitate triadic communication further

To facilitate a good conversation where everyone feels heard

Some insights into how this communication can be made smoother

How to manage complex communication situations around death and dying for young patients with young people and in a triadic situation

Build confidence and gain some strategies to ensure all are heard, feel respected and good foundation of therapeutic relationship made to help future interactions

I hope to learn how to communicate effectively in a triadic setting and improve my confidence at communicating with TYAs and the third person when there is a 3^rd^ person present

Effective communication skills to ensure that the patient feels heard and seen whilst delivering clear and safe information to enable them to make well informed choices

Develop on existing knowledge and develop new techniques

Better understanding of triadic communication and its application

Evaluation from teaching one

1. Please rate the teaching by relevance and learning.

2. How likely would you be to recommend this teaching to a colleague?

3. What went well?

Very thought provoking, break out room discussions.

Understanding the perspective of parents and how to adjust the way of broaching situations and how to support both discussions.

Break out groups and separate discussions. Videos with analysis then seeing the video again and analysing improvement. It felt like it consolidated learning better, like advanced communication skills training without us being videoed! Pre session work was helpful and made the case study better as concentrating on options and answer knowing the theory/what is expected by law.

Case studies were illustrative.

Very interactive and thought provoking.

Very well interactive and punctual.

4. What would you change?

Nothing.

Nothing. Agenda setting at the beginning of a clinic will be super helpful.

Nothing about course or content…just find TEAMS easier to navigate.

It was quite short! I was really enjoying it. Maybe looking at those articles together, drawing out some pertinent points would be good.

Have it in person.

5. Write down at least one learning point that you will incorporate into your clinical practice.

Agenda setting at the beginning of a clinic will be super helpful.

The explore, explain, explore.

Can’t’ only say one thing! Introducing self and asking YP to introduce supporter, asking what they expect to happen, set agenda and check it is ok with all.

Setting out agenda early on in the discussion, I definitely don’t do that, and I can see how useful it would be.

Importance of time alone.

Approach of alone time with TYA patient

6. Any other comments about the teaching overall - we welcome detailed descriptive feedback. Thank you

Really helpful to think about empowering the young person but not removing parental/partner, importance within the discussions.

Very well structured and informative around complex discussions and ways to approach them. Looking at examples that are not all wrong but maybe need adaptation for patients and families

Really good training that should be part of induction for TYA staff

Interaction was good, splitting into groups worked well. Thank you!

Gave clear information, great delivery and interactive, everyone had time to give their thoughts and interpretation

Evaluation from teaching two

1. Please rate the teaching by learning and relevance.

2. How likely would you be to recommend to colleagues?

3. What went well?

Examples and interactions

Really interactive, great case studies

Reinforced current communication and gave additional suggestions for speaking with young people

It was really useful exploring key concepts in triadic communication

Use of videos, small group conversations and chat function. Small group of (6) of us learning made it interactive and engaging.

Good to use chat for communication during the presentation. All felt very safe place to express opinion, answer questions etc. Very good content. East to follow and engaging. Good use of separate rooms and groups for discussion.

4. What would you change?

I’m outpatient based so perhaps ethical situation for long term patients not necessarily palliative – although similar strategies can be incorporated! Also younger young people scenario would be great as slightly different issues I believe.

Could make this into a 1-day teaching session in the long term

Nothing

Whilst it has been useful watching the videos, it would be even more useful to include elements of simulated role play (where we would practice talking to actors acting as the patient and third person)

Nothing comes to mind. I expect further sessions in the future would help embed the learning.

5. Write down at least one learning point that you will take into your clinical practice?

The importance of offering time alone as I am a physio and I’m mainly paediatrics it is not something I do much!

Ensuring young people have their own space to talk

Offering and facilitating one-to-one discussions with young people

Explaining WHY time alone with the TYA is important both for the TYA and the third person.

Centring the consultation around the TYA rather than the third person

Prioritising time alone and how to navigate that happening

Ensuring space for the young person to have a separate discussion away from the supporter

6. Any other comments

Really well presented and involving MDT and not too many people.

Great, thanks

No

Thank you so much for organising this teaching! It’s been really useful!

Really helpful. I wonder if it is more difficult to practise when working in a designated hospital with wider teams that don’t work with TYAs regularly. Have to persuade doctors etc to make time for young people alone.

Very good teaching session. Relevant and useful.
