# Supplementary material for: Co-designing a prototype teaching intervention on triadic communication for healthcare professionals working with young people with cancer
Source: PEC Innov. 2026 Jul 14;9:100492. doi: 10.1016/j.pecinn.2026.100492 (PMC13449821; doi:10.1016/j.pecinn.2026.100492)
Supplement: Supplementary file 3 [file mmc3.docx]

COREQ checklist

| **Checklist item** | Page reported |
| --- | --- |
| **Domain 1: Research team and reﬂexivity**  *Personal Characteristics*   1. Interviewer/facilitator   Which author/s conducted the interview or focus group? |  |
| 2. Credentials   What were the researcher’s credentials? E.g. PhD, MD |  |
| 3. Occupation   What was their occupation at the time of the study? |  |
| 4. Gender   Was the researcher male or female? |  |
| 5. Experience and training   What experience or training did the researcher have? |  |
| *Relationship with participants*   6. Relationship established   Was a relationship established prior to study commencement? |  |
| 7. Participant knowledge of the interviewer   What did the participants know about the researcher? |  |
| 8. Interviewer characteristics   What characteristics were reported about the interviewer/facilitator? |  |
| **Domain 2: study design**  *Theoretical framework*   9. Methodological orientation and Theory   What methodological orientation was stated to underpin the study? |  |
| *Participant selection*   10. Sampling   How were participants selected? e.g. purposive, convenience, consecutive, snowball |  |
| 11. Method of approach   How were participants approached? e.g. face-to-face, telephone, mail, email |  |
| 12. Sample size   How many participants were in the study? |  |
| 13. Non-participation   How many people refused to participate or dropped out? Reasons? |  |
| *Setting*   14. Setting of data collection   Where was the data collected? e.g. home, clinic, workplace |  |
| 15. Presence of non-participants   Was anyone else present besides the participants and researchers? |  |
| 16. Description of sample   What are the important characteristics of the sample? e.g. demographic data, date |  |
| *Data collection*   17. Interview guide   Were questions, prompts, guides provided by the authors? Was it pilot tested? | Supplementary files 1-3 |
| 18. Repeat interviews   Were repeat interviews carried out? If yes, how many? | N/A |
| 19. Audio/visual recording   Did the research use audio or visual recording to collect the data? |  |
| 20. Field notes   Were ﬁeld notes made during and/or after the interview or focus group? |  |
| 21. Duration   What was the duration of the interviews or focus group? |  |
| 22. Data saturation   Was data saturation discussed? |  |
| 23. Transcripts returned   Were transcripts returned to participants for comment and/or correction? | N/A |
| **Domain 3: analysis and ﬁndings**  *Data analysis*   24. Number of data coders   How many data coders coded the data? |  |
| 25. Description of the coding tree   Did authors provide a description of the coding tree? |  |
| 26. Derivation of themes   Were themes identiﬁed in advance or derived from the data? |  |
| 27. Software   What software, if applicable, was used to manage the data? |  |
| 28. Participant checking   Did participants provide feedback on the ﬁndings? | N/A |
| *Reporting*   29. Quotations presented   Were participant quotations presented to illustrate the themes / ﬁndings? Was each quotation identiﬁed? e.g. participant number |  |
| 30. Data and ﬁndings consistent   Was there consistency between the data presented and the ﬁndings? |  |
| 31. Clarity of major themes   Were major themes clearly presented in the ﬁndings? |  |
| 32. Clarity of minor themes   Is there a description of diverse cases or discussion of minor themes? | N/A |
