# Supplementary material for: Co-designing a prototype teaching intervention on triadic communication for healthcare professionals working with young people with cancer
Source: PEC Innov. 2026 Jul 14;9:100492. doi: 10.1016/j.pecinn.2026.100492 (PMC13449821; doi:10.1016/j.pecinn.2026.100492)
Supplement: Supplementary file 4 [file mmc4.docx]

CRe-DEPTH – Criteria for describing and evaluating training interventions in healthcare professions

| **Criteria** | **Page number** |
| --- | --- |
| *Development of the training* | |
| 1 Description of the aim or objectives of the training | Table 1 |
| 2 Description of the underlying theoretical framework | Page 11 |
| 3 Description of the development process | Page 3 |
| 4 Description of the target population and setting of the training | Page 4 |
| 5 Description of the educational resources | Supplementary material scheme of work |
| *Characteristics of the training* | |
| 6 Description of the content of the training | Table 3 and supplementary material scheme of work |
| 7 Description of the format | Page 6 and supplementary material scheme of work |
| 8 Description of the didactic methods of training | Supplementary material scheme of work |
| 9 Description of tailoring the training | Supplementary material scheme of work |
| *Characteristics of the providers* | |
| 10 Description of the providers of the training | Page 5 |
| *Assessment of the training outcomes* | |
| 11 Description of the measured outcomes | Supplementary material – post teaching questionnaire. |
| 12 Description of the applied assessment method | n/a |
